# Automated measurement of total kidney volume from 3D ultrasound images of patients affected by polycystic kidney disease and comparison to MR measurements

**DOI:** 10.1007/s00261-022-03521-5

**Published:** 2022-04-27

**Authors:** Jaidip M. Jagtap, Adriana V. Gregory, Heather L. Homes, Darryl E. Wright, Marie E. Edwards, Zeynettin Akkus, Bradley J. Erickson, Timothy L. Kline

**Affiliations:** 1 Department of Radiology, Mayo Clinic, 200 First St. SW, Rochester, MN 55905 USA; 2 Division of Nephrology and Hypertension, Mayo Clinic, Rochester, MN 55905 USA

**Keywords:** Ultrasound images, U-Net, ADPKD, Kidney segmentation, MRI, TKV

## Abstract

**Purpose:**

Total kidney volume (TKV) is the most important imaging biomarker for quantifying the severity of autosomal-dominant polycystic kidney disease (ADPKD). 3D ultrasound (US) can accurately measure kidney volume compared to 2D US; however, manual segmentation is tedious and requires expert annotators. We investigated a deep learning-based approach for automated segmentation of TKV from 3D US in ADPKD patients.

**Method:**

We used axially acquired 3D US-kidney images in 22 ADPKD patients where each patient and each kidney were scanned three times, resulting in 132 scans that were manually segmented. We trained a convolutional neural network to segment the whole kidney and measure TKV. All patients were subsequently imaged with MRI for measurement comparison.

**Results:**

Our method automatically segmented polycystic kidneys in 3D US images obtaining an average Dice coefficient of 0.80 on the test dataset. The kidney volume measurement compared with linear regression coefficient and bias from human tracing were *R*^2^ = 0.81, and − 4.42%, and between AI and reference standard were *R*^2^ = 0.93, and − 4.12%, respectively. MRI and US measured kidney volumes had *R*^2^ = 0.84 and a bias of 7.47%.

**Conclusion:**

This is the first study applying deep learning to 3D US in ADPKD. Our method shows promising performance for auto-segmentation of kidneys using 3D US to measure TKV, close to human tracing and MRI measurement. This imaging and analysis method may be useful in a number of settings, including pediatric imaging, clinical studies, and longitudinal tracking of patient disease progression.

**Graphical abstract:**

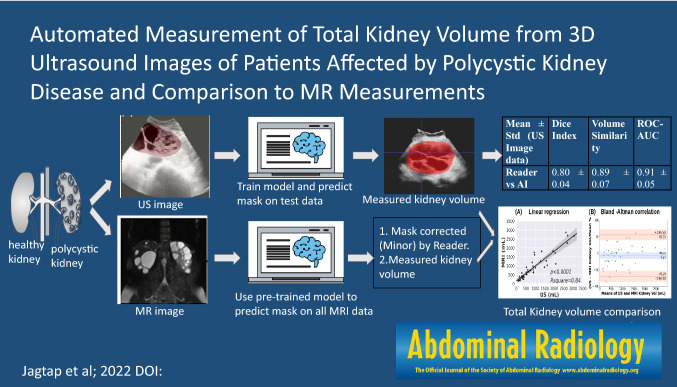

**Supplementary Information:**

The online version contains supplementary material available at 10.1007/s00261-022-03521-5.

## Introduction

Polycystic kidney disease (PKD) is a genetic disorder in which cysts develop within the kidneys, causing kidneys to enlarge and lose function over time[1]. Nine out of ten people with PKD have the autosomal dominant form (ADPKD) [2, 3]. In ADPKD, cysts develop primarily in the kidneys but can also be present in other organs, like the liver. Currently, there are ~ 140,000 people diagnosed with ADPKD in the United States [4]. Over time, kidney and liver volumes steadily increase, resulting in renal function decline [5, 6]. In particular, renal elasticity is associated with kidney function [7]. There is no cure for PKD, but dialysis, kidney transplant, blood pressure medication, and surgical removal of cysts are treatment options. If diagnosed and monitored at an early stage, better treatment options are possible.

Measuring kidney and liver volumes are some of the most important biomarkers in quantifying the severity of ADPKD and are used in clinical decision making [8–10]. Also, many studies found that TKV, along with age, height, and estimated glomerular filtration rate (eGFR) are useful prognostic biomarkers to predict renal function decline [11–13]. Bae et al*.* [14] reported MRI-based kidney volume measurement in ADPKD by manually segmenting the slices in kidney volume. However, annotating each slice for volume measurement is laborious, and to overcome this, many researchers have recently utilized AI for automatic kidney segmentation [15–19]. Keshwani et al. [20] used a 3D convolutional neural network (CNN) for automated kidney segmentation in CT scans. Sharma et al*.* [16] used a CNN with a visual geometry group (VGG) like structure for automated kidney segmentation from the CT dataset of ADPKD. In MR images, Mu et al*.* used a 3D V-Net model for automated kidney segmentation in ADPKD data [21]. van Gastel et al*.* [22] used semantic segmentation for automated measurement of both kidney and liver volumes in MR images of patients affected by ADPKD. Kline and his group used instance segmentation [23] and semantic segmentation [24] for kidney cyst segmentation in T2-weighted MR images of ADPKD patients for total cyst volume.

Apart from MRI and CT imaging, ultrasound (US) imaging is popular and widely used to diagnose acute and chronic kidney diseases [25, 26]. Kuo et al*.* [27] used 2D US images to perform automated classification of kidney images using ResNet, to determine chronic disease status but did not use segmentation. Mahmud et al*.* [28] used vector graphic detection image analysis for kidney and cyst boundary detection from 2D images along with various texture analyses, filtering, and patches to detect kidney boundaries from limited data. However, no further updates were found with this study on large data, or no development was reported by any other group. Imaging features computed from US data using deep CNNs improved the classification of children with congenital abnormalities of the kidney and urinary tract and controls [29]. However, the computation of these anatomic measures typically involves manual or semi-automatic segmentation of kidneys in US images, requiring multiple human annotators, increasing inter-operator variability, reducing reliability, and limiting utility in clinical medicine. Automatic kidney segmentation in US images with AI has not progressed recently. US images have irregular scan plane acquisition and low-image contrast, making it difficult to segment the kidney accurately. Image quality, image size and magnification, gain, nonuniform intensity and contrast, and human variability in moving the US probe contribute to the challenges faced in segmenting US images [30]. Having an extensive dataset could help overcome the artifacts from individual images and result in better model training. With a small dataset, data augmentation is a technique used to introduce variability in training data, which helps model training [31].

Automatic and reliable kidney segmentation from US images would improve precision and efficiency in many clinical conditions, including congenital renal disease, renal mass detection, and kidney stones. Very few studies have reported US-kidney segmentation using deep learning. Wu et al*.* [32] reported cascaded fully convolutional DenseNet for automatic kidney segmentation of 2D US images. The mean intersection over union for FC-DenseNet was improved slightly with cascaded FC-DenseNet on 461 images of 68 patients. Yin et al*. *[33] performed automatic kidney segmentation of 2D US images using a pre-trained VGG16 model and weights (i.e., a transfer learning-based approach) and subsequent boundary distance regression (Bnet) and pixel-wise classification with a Deeplab network. The algorithm was trained on 289 images, but this study was limited to the largest 2D sagittal image from the whole kidney. Because a single 2D image does not produce an accurate measurement of kidney volume, particularly in a disease-like ADPKD where cysts often produce a very irregular shape, a 3D US scan is vital to obtain accurate TKV measurements. The variability severely degrades the performance of AI models in kidney shape, imaging protocol variability, instrument resolution, and imaging field of view. Recently, Breysem et al*.* used 3D US as an alternative to MRI for measuring renal volume in children with ADPKD and found 2D US measurements had significantly lower kidney volume than the 3D US, and 3D US measurements of TKV were close to MRI measurement [34]. Hence, there is a need for further development of US-based kidney imaging and segmentation to understand the problems and improve the performance of the AI models in segmentation.

In this study, to mitigate some of the aforementioned issues with 2D images, we acquired 3D US images using an electromagnetic tracker attached to the US probe. This tracks the position and angle of a probe in space and results in a 3D stack of aligned 2D images. TKV was calculated from the 3D images of the kidneys. We trained a U-Net model to segment kidneys using 3D US images, allowing us to measure TKV automatically. All participants in this study were also imaged by MRI, and kidney volume measured using MRI serves as the reference standard. TKV measurements using AI-empowered 3D US could be an alternative approach to where MRI is challenging for diagnosing and monitoring ADPKD patients.

## Methods and materials

The Institutional Review Board of Mayo Clinic, Rochester, USA, approved this study protocol, and informed consent was obtained from each participant. Patient data were anonymized before use. Table 1 shows the demographic information of the 22 patients and computed body mass index (BMI). Fourteen participants in the study were women (64%) and eight were men (36%). The mean and median age of the study cohort was 51 (min = 28, max = 70) and 48 years, respectively. The mean and median patient height were 1.71 and 1.68, respectively. Five patients had a normal BMI (i.e., BMI between 18.5 and 24.9 kg/m^2^), eleven patients were overweight (i.e., BMI between 25 and 29.9 kg/m^2^), and six patients were obese (i.e., BMI of 30 kg/m^2^ or higher).

**Table 1:** The demographics from the ADPKD study cohort

Study ID	Sex	Age	Height (m)	Body Mass Index (kg/m^2^)
PKD_003	M	46	1.89	27.80
PKD_004	M	50	1.81	32.17
PKD_005	F	60	1.66	28.12
PKD_006	M	55	1.91	23.27
PKD_008	F	32	1.63	22.96
PKD_009	M	75	1.72	32.89
PKD_010	F	69	1.63	20.81
PKD_011	F	56	1.65	30.89
PKD_012	F	45	1.63	27.93
PKD_013	M	37	1.75	27.84
PKD_014	F	60	1.60	32.90
PKD_015	F	40	1.69	27.99
PKD_016	F	42	1.65	25.49
PKD_017	M	43	1.88	28.52
PKD_018	F	35	1.71	27.15
PKD_019	M	40	1.78	28.25
PKD_020	F	70	1.75	24.37
PKD_021	M	70	1.86	32.89
PKD_024	F	70	1.56	27.20
PKD_026	F	38	1.62	31.36
PKD_027	F	28	1.66	23.22
PKD_028	F	62	1.65	29.90

### Ultrasound imaging

We used axially acquired 3D US-kidney images in 22 ADPKD patients. Each patient had both kidneys imaged three times, resulting in 132 (22 patients × (3 scans of left kidney + 3 scans of the right kidney)) image sets. Images were acquired with a Philips EPIQ 7 system using the C5-1 curved linear probe with broadband 1–5 MHz frequency range and electromagnetic probe positioning system. 2D B-mode imaging was selected for this study. The resolution/speed settings were adjusted in order to improve the resolution of images. The time-gain-compensation and image gain were optimized per patient, where the image gain ranged from 54 to 68%. A freehand sweep with an electromagnetic tracker attached to the probe for recording probe orientation was used to build a 3D volume stack from 2D cross-sectional images of kidneys aligned based on probe orientation. The image size was either 256 × 256 or 512 × 512; the frames (Z dimension) varied from 300 to 700. The axially acquired DICOM format US images were transformed into NIfTI with *SimpleITK* and Python. Semi-automated in-house-developed software customized for US images was used to annotate the kidney at every frame [35]. Two experienced readers performed the manual tracing for kidney segmentation on the 3D US images. Reader1 (A.V.G.) performed annotations for all the data, whereas Reader2 (H.L.H.) performed annotations on a subset of 54 scans (9 patients × 6 scans) to measure inter-rater agreement.

### Deep learning model

Each image set was zero-padded or cropped to 320 × 320 × Z, such that the entire kidney was always fully included in the volume. In the scanned sequence, many frames from the start and end of the sequence did not contain any data, and such blank (zero intensity) frames were omitted in order to avoid data imbalance issues. The threshold was chosen to include frames that had at least 20% non-zero pixels in the frame. The train:validation:test split was 15:2:5 at the patient level, resulting in 90:12:30 scans respectively, where each scan consisted of a series of 300–700 slices. Data augmentation, including ± 15° rotation and random elastic deformation, resulted in a three times increase in training dataset size and was performed to introduce variability in training data for better model generalization. We observed that random flip worsened the model performance, and Gaussian noise had minimal or no effect on model performance (and was, thus, not used in the final training experiments).

A transfer learning approach was used in this study. The architecture and weights from our previously reported 2D U-Net model [19] were used to train on the US data for kidney segmentation (Schematic of U-Net structure presented in Supplementary data, Fig S1). A 6-layer 2D U-Net model (filters varied from 32 to 1024, kernel size varied from (7,7) to (5,5) and then (3,3) at the base layer and gradually increased back to (7,7) at the top level of decoders) was trained to segment the whole kidney in US images. The input data shape (3, 256, 256) was provided to the model, and a user-created mask on the central slice was used. The sigmoid activation was applied to the last layer of the U-Net. The learning rate was 10^–6^, and the loss function was the Dice loss. Each model was trained for 200 epochs. The model was implemented in Keras with the TensorFlow backend and trained on an Nvidia V100 (32 GB memory).

### MRI imaging

All 22 patients imaged with the US were also imaged with MRI that included 3–4 mm thick slices obtained in the coronal plane with T2-weighting and fat saturation [19]. There were no separate groups assigned to one image technique or the other. We applied our previously described model [19] to all 22 cases as the automated MRI measurement. These segmented kidney masks were further quality checked by an expert image analyst (A.V.G.). The mask was corrected if needed to finalize the segmentation and measure kidney volume.

### ADPKD classification

The Mayo ADPKD classification tool [36, 37] was applied for ADPKD group classification to both the MRI and the US images. This tool uses the information of patient age, TKV, and height of the patient.

## Results

### Data processing

**Fig. 1:**
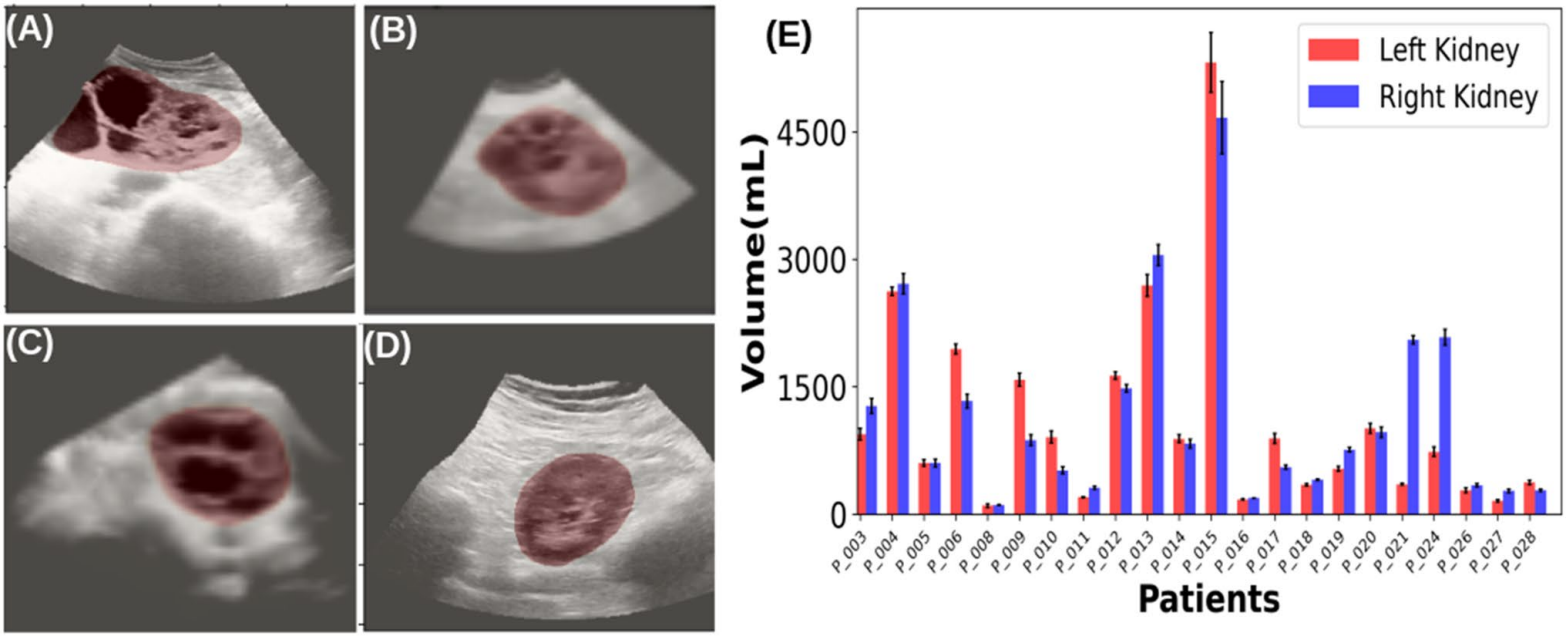
US images of the kidney. These demonstrate common challenges including **a** large kidney size, **b** small field of view, **c** contrast variation, and **d** centered kidney. **e** shows the left and right kidney US volume measurements (mean and deviation for each subject

US-kidney images have variable intensity, shape, and size, as demonstrated in Fig. 1a–d. Unlike MRI and CT, US-imaging scans each kidney individually due to probe field of view limitations. In some cases, the field of view may not be sufficient for imaging large kidneys (Fig. 1a). The variability of kidney measurements in the left and right kidneys for each subject was measured as displayed in Fig. 1e. The mean and standard deviation were obtained from three scans from each individual kidney.

### U-Net model training and test data evaluation

**Fig. 2.**
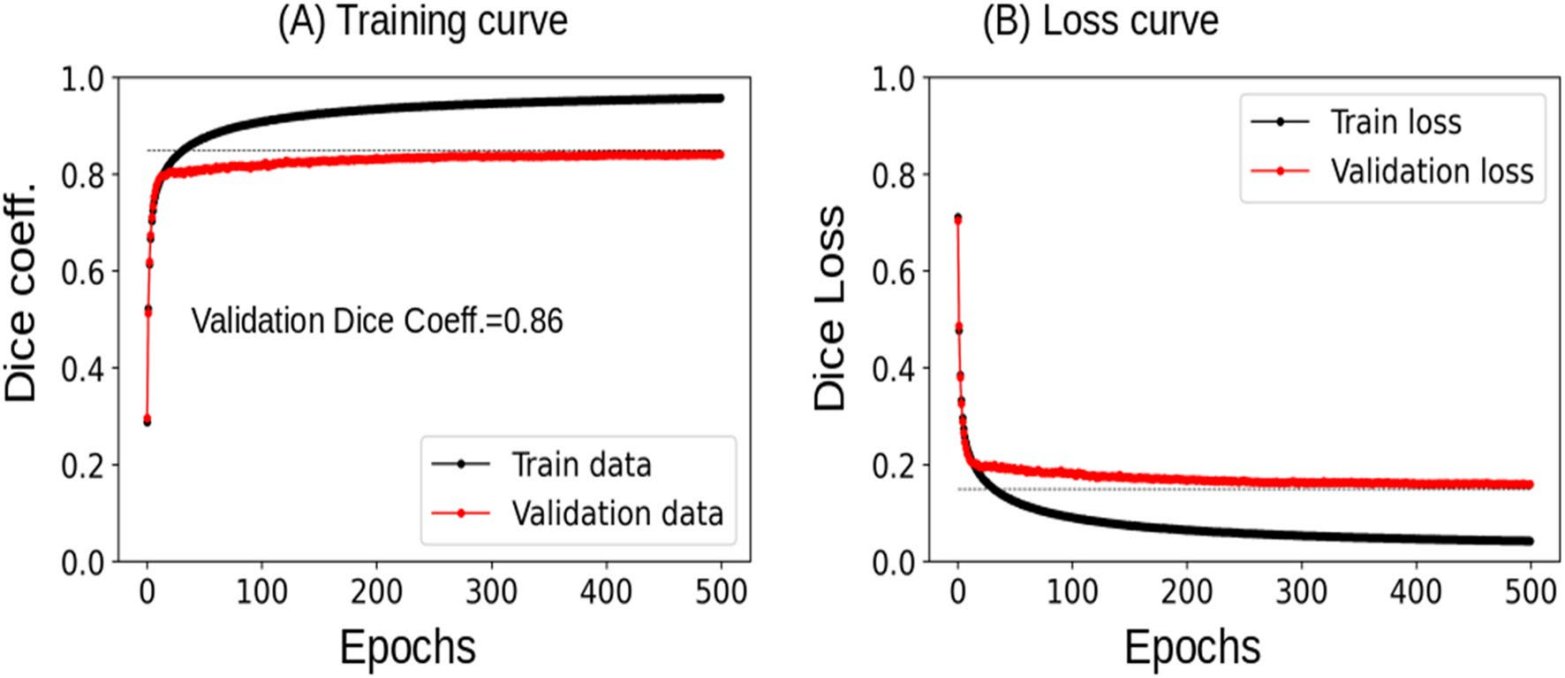
2D U-Net model trained with the pre-trained weights **a** training curve and **b** loss curve

The U-Net model with pre-trained weights (from the MRI model) was trained on 90 scans (15 patients × 2 kidneys × 3 scans) and validated with 12 scans (2 patients × 2 kidneys × 3 scans) as demonstrated in Fig. 2. The U-Net model trained with transfer learning from pre-trained weights achieved a Dice Similarity Coefficient (DSC) on the validation data of 0.86 (Fig. 2a). The trained U-Net model was used to predict kidney segmentations on the hold-out test dataset for further analysis. On the test dataset of 30 scans (5 patients × 6 scans), the model had a 0.80 DSC, 0.67 Jaccard Index, 0.89 volume similarity (VS), 0.83 Matthews correlation coefficient (MCC), 20.65 average Hausdorff distance with 95 percentile of maximum distance (HD-95%), and 0.91 AUC, respectively. Table 2 has tabulated these parameters as mean ± standard deviation from 30 scans along with false negatives (FN), and the comparison of model prediction against Reader1 and Reader2. The last row compares the metric parameters from Reader1 and Reader2.

**Table 2:** Model prediction and comparison with Reader1 and Reader2

Mean ± Std	Dice Index	Jaccard Index	FN	MCC	HD-95%	VS	ROC-AUC
Reader1 vs AI	0.80 ± 0.05	0.67 ± 0.07	0.17 ± 0.11	0.83 ± 0.09	20.65 ± 6.58	0.89 ± 0.07	0.91 ± 0.05
Reader2 vs AI	0.79 ± 0.09	0.66 ± 0.11	0.17 ± 0.08	0.82 ± 0.08	19.23 ± 5.88	0.92 ± 0.07	0.91 ± 0.04
Reader1 vs Reader2	0.77 ± 0.13	0.64 ± 0.16	0.21 ± 0.17	0.80 ± 0.13	24.33 ± 12.76	0.86 ± 0.15	0.89 ± 0.08

**Fig. 3:**
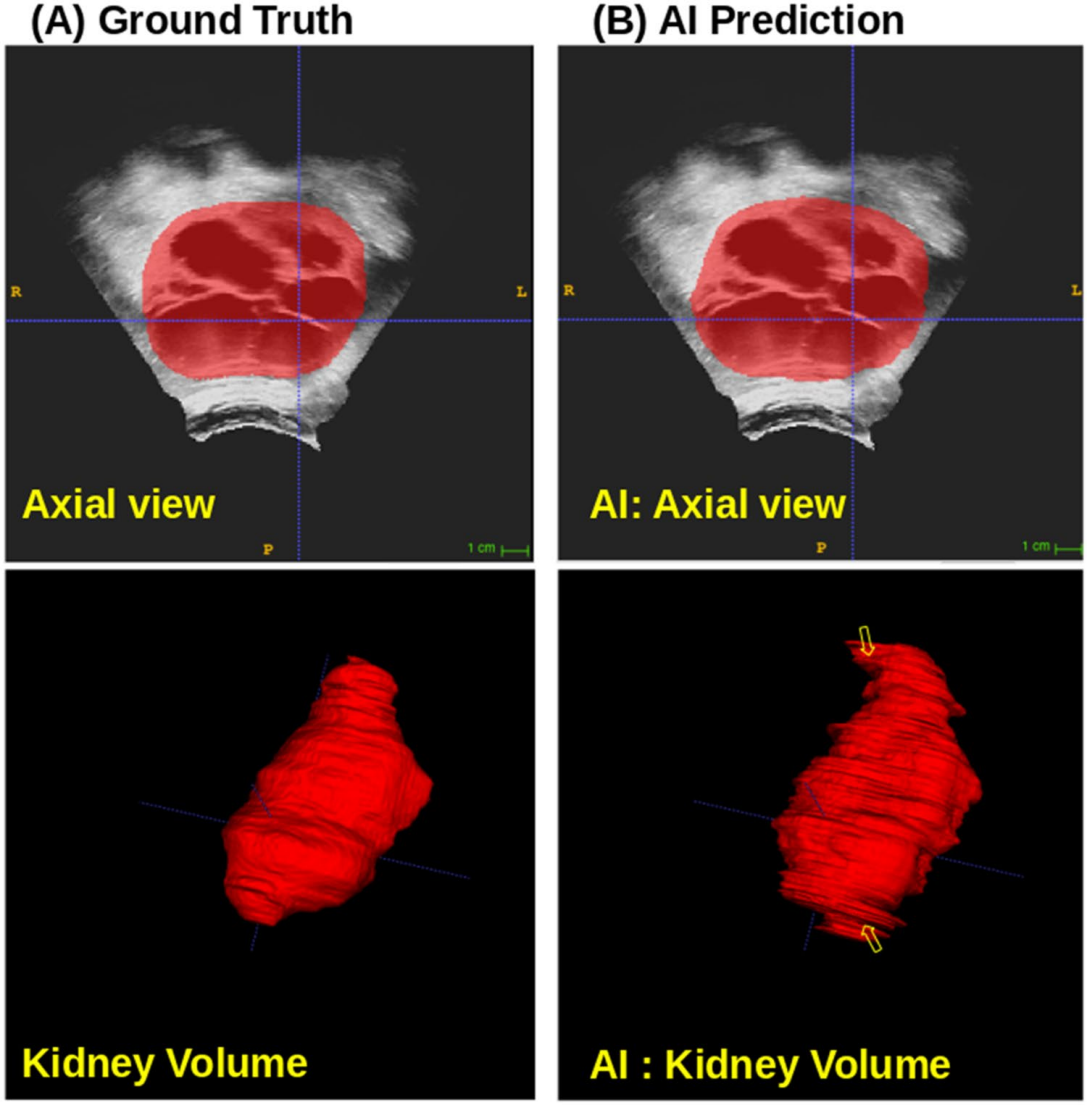
A typical example of AI model-based whole kidney prediction compared with ground truth annotated whole kidney

Further, the DSC was calculated slice-wise to see the effect of kidney shape and size contribution in class imbalance and impact on the AI model performance. The slice number containing the whole kidney was split into 10 segments. The DSC was calculated for each segment of the kidney, as demonstrated in Table 3, for Reader1 vs. Reader2 and Reader1 vs AI prediction. The manual annotations and AI-based automated kidney region visualizations are shown in Fig. 3. The values in bold represent that AI performs similar to or better than human tracing in slices from the largest area of the kidney.

**Table 3:** Dice score at various slices in the kidney

Slice contribution	0–10% (Start)	10–20%	20–30%	30–40%	40–50%	50–60%	60–70%	70–80%	80–90%	90–100% (End)
Reader1 vs Reader2 (DSC)	0.53	0.73	0.82	0.83	0.84	0.82	0.79	0.74	0.68	0.47
Reader1 vs AI (DSC)	0.48	0.74	**0.83**	**0.85**	**0.85**	**0.85**	**0.87**	**0.85**	0.75	0.46

We also compared our model performance with other state-of-the-art U-Net models having backbones of VGG16, EfficientNet-B0, DensNet101, and ResNet50, as demonstrated in Supplementary data, Table S1. We chose these base models having trainable parameters close to our proposed model, ~ 18 M. The baseline U-Net with various backbone models was trained on the same training data and validation data for comparison. Pre-trained weights from ‘imagenet’ were used in training.

### Inter-reader and intra-scan variability

**Fig. 4:**
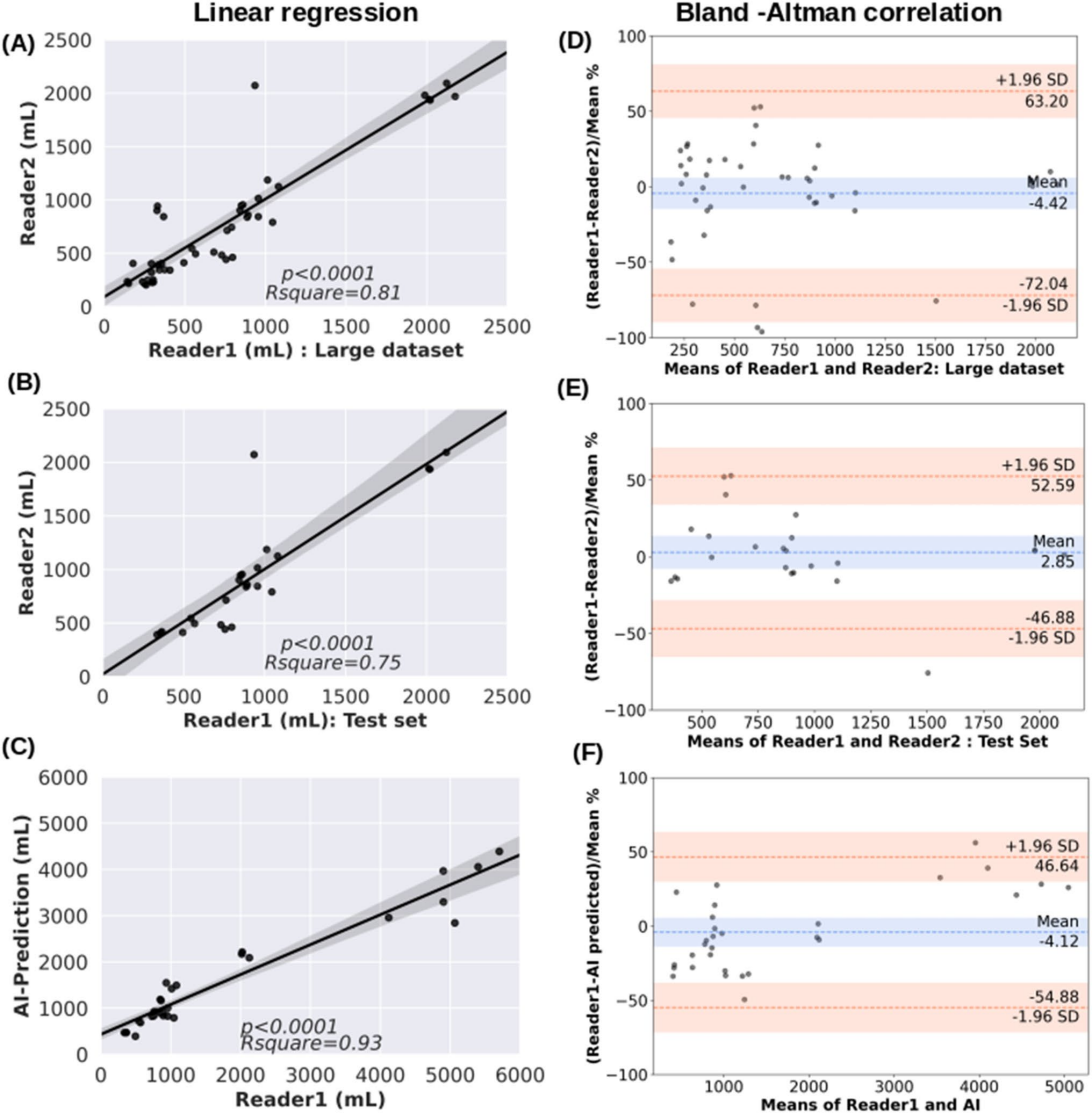
Inter-reader variability and Bland–Altman correlation among readers annotated kidney volume and versus AI model predicted kidney volumes

Two medical imaging analyst experts annotated kidneys on 3D US images, which were used to calculate each reader’s agreement in kidney segmentation. Figure 4 shows the inter-reader correlation and a reader’s comparison against AI-predicted kidney volume with Bland–Altman correlation methods. The Bland–Altman plot displays the differences among measurements on the same scale in percentage ((method1 − method2)/mean %), which is useful due to the range of variation in kidney volume present in data. The first row shows the results for inter-reader observations of Reader1 and Reader2 from 54 scans which had an *R*^2^ of 0.81 in linear regression (Fig. 4a), whereas Bland–Altman shows the bias (mean difference) − 4.42%. The bias is significant because the line of equality is not in the 95% confidence interval. The agreement limits were from − 72.04 to 62.20% (Fig. 4d). A subset of 24 scans used in the test dataset and have both readers tracing were compared in the second-row as Reader1 vs. Reader2, reduced the linear regression coefficient to *R*^2^ = 0.75 due to significantly different volume for one scan in a small dataset (Fig. 4b), where the bias was 2.85%. The agreement limits were from − 46.88 to 52.59% (Fig. 4e). The third row compares the Reader1 vs AI model for 30 scans test data correlation (Fig. 4c, f) where linear regression had *R*^2^ = 0.93 and bias = − 4.12% with the limits of agreements from − 54.88 to 46.64% in the Bland–Altman analysis.

The interscan variability was calculated by subtracting kidney volume from the mean of three scans and then taking the average of the absolute differences, which was found to be ~ 56 mL (interscan variability plot Supplementary data, Fig S2).

### MRI vs US correlation

**Fig. 5:**
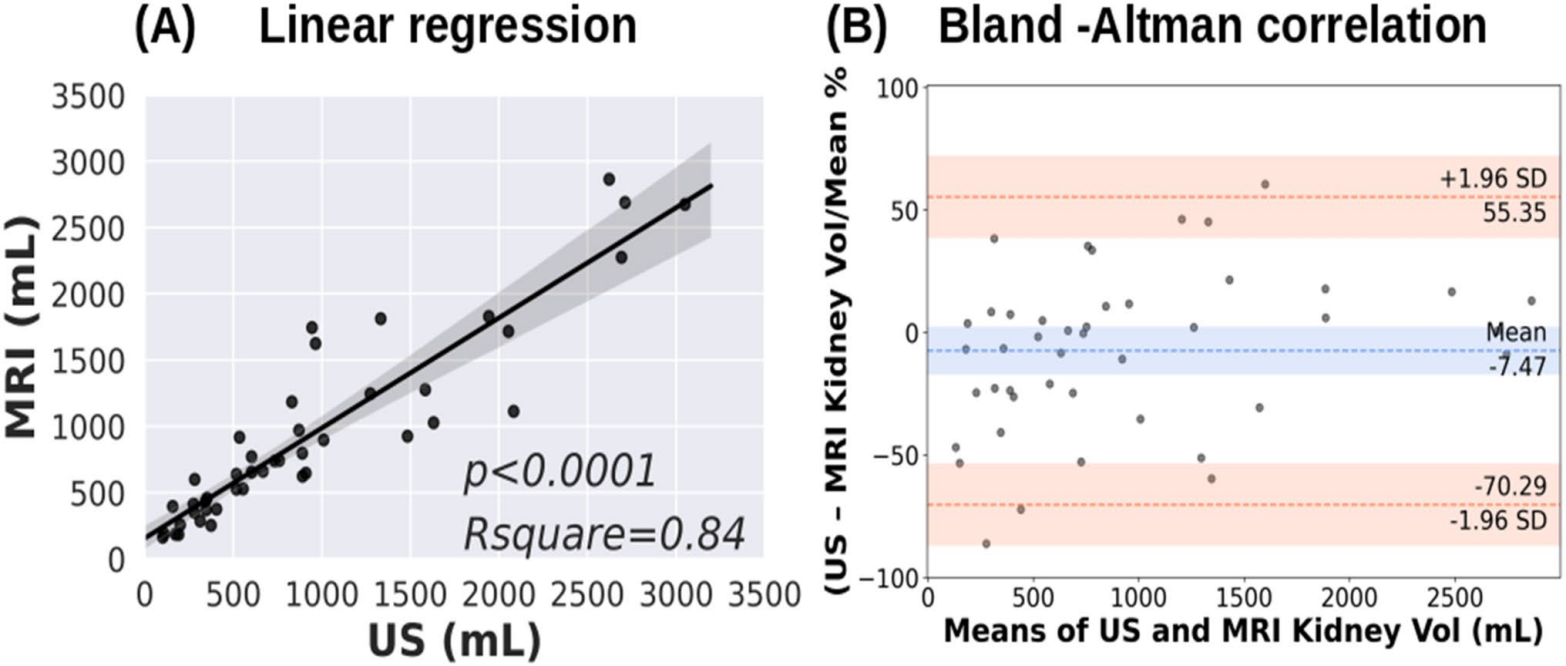
Total kidney volume comparison by linear regression and Bland Altman correlation for MR and US methods

Human-corrected kidney volumes (left and right kidney separately) from MRI images were compared with manually annotated kidney volumes (left, right kidney volumes separately) in 3D US images at the patient level. Figure 5 demonstrates MRI and US measured kidney volumes comparisons gave a linear regression coefficient *R*^2^ = 0.84 and bias of 7.47% with the limit of agreement from − 70.29 to 55.35% by Bland–Altman analysis.

### ADPKD group classification

Mayo Clinic’s ADPKD classification tool [36] was applied to all 22 patients to compare US and MRI performance, as demonstrated in Table 4. Also, the ADPKD classification was performed separately by considering the test dataset only and compared classification across the US and MRI methods. The groups 1A to 1E indicate increasing severity of cases in ADPKD classification. In the US method, both kidney volumes were added to get total kidney volume and then averaged over three observations to consider total kidney volume per patient. Out of 22 patients, both MRI and US classified three patients of 1A group in common, whereas from US-manual measurement, one extra got added to each of 1B and 1C from 1C and 1D, respectively. In contrast, one patient measured higher kidney volume by the US and shifted from group 1D to 1E. On the test dataset (5 patients), the ADPKD classification tool shows good agreement in 4 patients for US-manual vs. AI prediction. Whereas, when comparing the US measurement to MRI measurement, AI prediction marginally performed better and closer to MRI measurements than manually annotated kidney volumes from US groups. Both the groups, manual tracing and AI prediction, in the US-based kidney volume measure higher volume in polycystic liver disease (PLD) cases due to difficulty in differentiating the liver from the kidney.

**Table 4:** ADPKD group classification and comparison between MRI and US classification

Manual tracing	1A	1B	1C	1D	1E	Total
MRI	3	5	8	4	2	22
US-manual	3	6	7	3	3	22
Test set	Patient1	Patient2	Patient3	Patient4	Patient5	Total
MRI	1C	1C	1D	1C	1B	5
US-manual	1C	1E (PLD)	1C	1B (under-segment)	1C	5
US-AI	1C	1E (PLD)	1C	1C	1C	5

## Discussion

Two-dimensional US is difficult to use for kidney volume measurement due to variability in how the operator moves and holds the probe, resulting in inconsistent image spacing and orientation. However, a recently developed 3D US device can be used to measure kidney volume and has the benefit of acquiring images with a high temporal resolution, which limits motion and other artifacts. Still, due to field of view limits, only a single kidney can be imaged at one time. Such images also have probe sensitivity, intensity variation, brightness, and time-gain compensation artifacts which must be addressed. For example, Fig. 1e demonstrates the average kidney volume from three scans, where PKD_021 and PKD_024 show a significantly larger right kidney than the left kidney. MRI also confirms similar differences in PKD_024 for left and right kidney volume. The cyst development and kidney swelling in ADPKD patients often result in increased kidney volume. However, in PKD_021, both the US Readers traced the right kidney larger than the left, which was contrary to MRI measurements. The image quality with intensity and contrast severely affected kidney tracing in some US images. This problem could be tackled by better handling of image acquisition protocol, modifying probe setting parameters based on patient demographic information.

The proposed model was trained with pre-trained weights that performed better on the validation data than the randomly initialized model. From the state-of-art U-Net models, ResNet achieved similar performance to our proposed model but needed 1.7 × more parameters and more time to train the model. Since pre-trained weights were from our previously published model from a large dataset of MRI images, 2000 + patients corresponding kidney segmentations, we chose to report the transfer learning model and perform prediction on the test dataset. Even though the AI model was trained on annotations received from Reader1, its performance was equally comparable to Reader2 annotations (Table 2). Table 3 demonstrates that the Dice score was reduced on the start and end frames from the kidney compared to the center frames from the kidney. This reduced DSC was primarily due to model under-performance where class imbalance was greater at the start and end frames in the kidney. Also, the contribution from overfitting (shown by arrows in 3B, False positives) resulted in a ~ 2% increase in volume, which is significantly smaller than the interobserver annotations DSC loss of ~ 23%. The interobserver score measured between Reader1 and Reader2 (Table 2) through the DSC was 0.77. The primary reason behind Reader’s disagreement was image quality which makes kidney boundary detection difficult. Although AI model performance looks impressive, we note that this is a small test dataset and may further improve with a larger dataset.

Yin et al*.* [33] reported a superior DSC performance on 2D US images, also for segmenting a single slice containing the largest kidney cross section in sagittal view. Further Breysem et al*.* [34] found that the 2D US volumetry was prone to underestimation, and 3D US measured more accurate kidney volume and was close to the MRI technique. We believe ours is the first study applying deep learning to 3D US from ADPKD. Further, the AI model results were compared to both human annotations and MRI as a reference standard.

Considering the artifacts in US images affecting the AI model performance, manual segmentation comparison on 54 kidney volume measurements helped understand the interobserver variability. The linear regression plot (Fig. 4a) of Reader1 and Reader2 displayed an interobserver correlation of *R*^2^ = 0.81, likely reflecting the lower tissue contrast observed in the US versus MRI [19]. The R^2^ coefficient was also low when Reader1 and Reader2 were compared on a small test dataset (Fig. 4b), mainly because a few observations significantly deviated. Figure 4c shows linear regression plots that help understand that Reader and AI prediction are correlated. Furthermore, the R^2^ may not be a good parameter to characterize performance when a small dataset is used. The 2nd column in Fig. 4 displays the Bland–Altman test, and the mean and bias were small when applied on a test dataset (Fig. 4e).

The volume of kidneys from the US (left and right kidney volumes averaged over three scans) method and MRI method were also highly correlated with a value of *R*^2^ = 0.84. The Bland–Altman bias of − 7.47% also confirms that the differences in US and MRI measurements are small, indicating the possibility that US imaging may be used to measure total kidney volume if needed for frequent monitoring of kidney volumes where MRI is challenging.

The ADPKD group classification tool [37] classified patients in ADPKD groups based on age, height, and total kidney volumes measured from MRI, US, and AI-predicted total kidney volumes. When the whole dataset of 22 patients was compared for MRI measurement vs US-manual traced measurement, one patient (PKD_015 in Fig. 1e) was classified into a higher risk group based on the US, from 1D to 1E. On review of that case, it was found that the case was difficult to segment and one of the Readers added the volume from some portions of the liver (the patient also had polycystic liver disease). Based on five patient test datasets, ADPKD group classification was not ideal between MRI and AI-US on three patients, including one PLD case. The interscan variability (Supplementary data, Fig S2), differences in three scans from the same kidney, calculated was ~ 56 mL. This volume has contributed to two patients whose kidney volume lies on the boundary of the ADPKD group classification threshold, to shift the ADPKD group. So the benefit of margin from interscan variability needs to be considered performing ADPKD group classification from US data.

Furthermore, on the right kidney of a PLD patient (PKD_015), AI predicted a 4 × smaller volume than Reader1 traced. Interestingly, AI predicted that this lower volume was similar to MRI measurement, which indicates AI could help correctly trace kidneys and avoid liver inclusion if trained with enough PLD patient data.

In the future, it could be interesting to add data from polycystic liver disease to the training data and perform segmentation on both kidneys and liver. In general, liver volumes are often calculated in patients affected by TKV and/or TLV. We believe that 3D US could also be applied to acquiring liver volumes, though this is beyond the scope of this present study. TKV is a strong biomarker of future renal insufficiency in ADPKD [38]. Various imaging techniques (MRI, CT, and US) and post-processing methods (stereology and ellipsoid-based measurements) are being used to determine TKV [38, 39]. Analysis with stereology is time consuming, whereas the ellipsoid method is easy for volume estimation. Since the kidney organ is located deep in the body, the US technique could easily show artifacts in images due to air/fluid/tissue distribution in the body. The 2D US images have in general poorer resolution than MRI. Therefore, MRI is the preferred method for accurate measurement of renal volume compared with both US and CT. However, recent developments with the 3D US could help improve results with US imaging. With a large dataset, the model performance would improve, and 3D US imaging may become incorporated into the clinical practice for PKD monitoring. Another potential of the US method could be in pediatric patients since it would be desirable to avoid MRI or CT imaging in that population.

## Conclusions

To the best of our knowledge, this is the first study to measure total kidney volume from 3D US images using deep learning. Our method shows promising segmentation performance for auto-segmentation of kidneys and calculating total kidney volume, close to human tracing, and measurement. We also compared its performance with MRI, and it achieved good performance, suggesting it may be useful in populations where MRI is more challenging, such as children.

## Supplementary Information

Below is the link to the electronic supplementary material.

Supplementary file1 (PDF 273 kb)

## Data Availability

The data underlying this article cannot be shared publicly due to the privacy of individuals that participated in the study. The data will be shared on reasonable request to the corresponding author.
