# Supplementary material for: Automated measurement of total kidney volume from 3D ultrasound images of patients affected by polycystic kidney disease and comparison to MR measurements
Source: Abdom Radiol (NY). 2022 Apr 27;47(7):2408–19. doi: 10.1007/s00261-022-03521-5 (PMC9226108; doi:10.1007/s00261-022-03521-5)
Supplement: Supplementary file 1 — Supplementary file1 (PDF 273 kb) [file 261_2022_3521_MOESM1_ESM.pdf]

Supplementary **Fig S1**: U-Net structure.

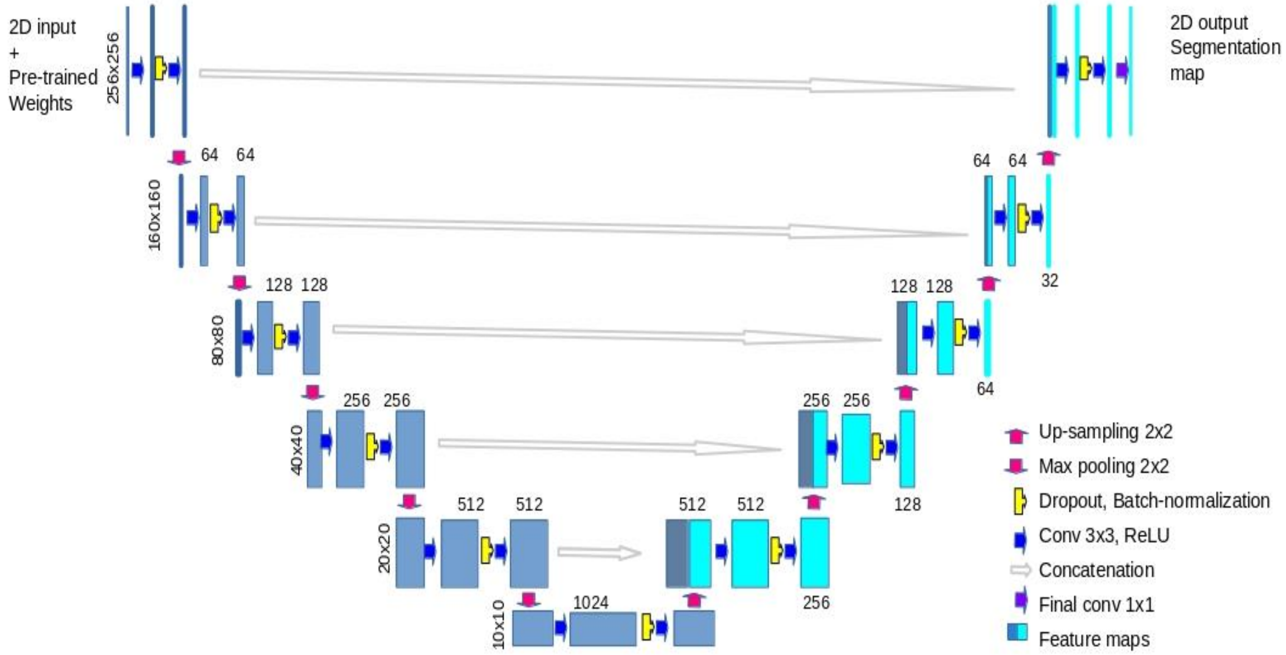

Supplementary **Fig S2**: Interscan variability plot.

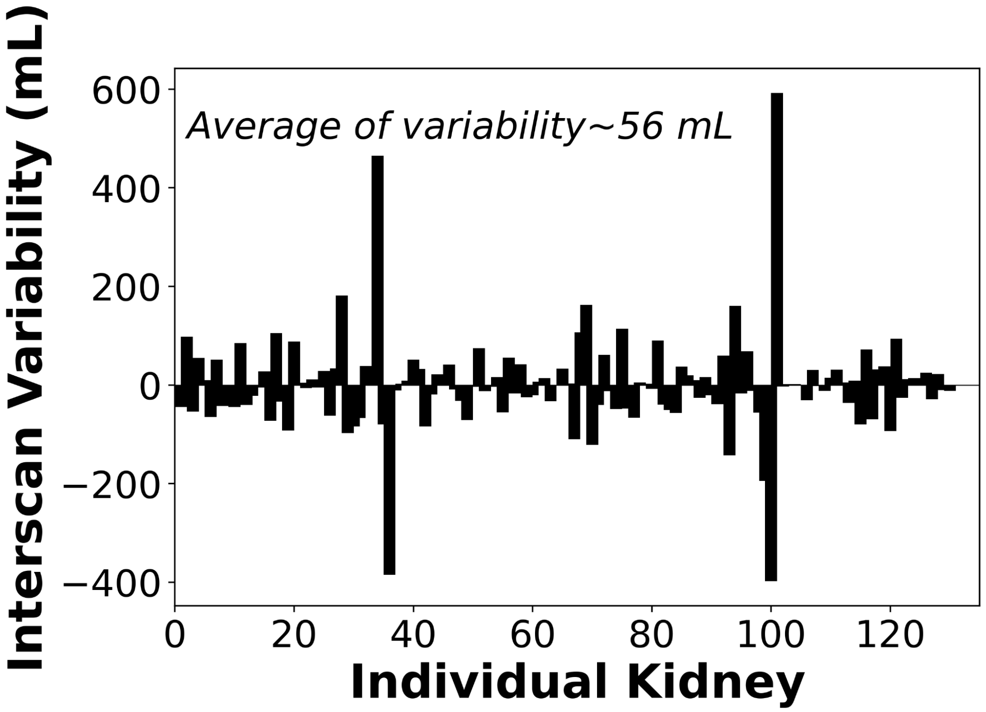

Appendix **Table S1**. Comparison of our proposed deep learning model performance with other U-Net models with various backbones structures.

**Model (input size)**	**Total Parameters**	**Val Dice**	**Test Dice**	**Test Accuracy**	**Test f1score**	**Test Precision**	**Test ROC-AUC**
**EfficientNetB0 (224x224x3)**	10,115,501	0.824	0.069	0.628	0.069	0.043	0.433
**DenseNet121 (224x224x3)**	12,144,977	0.831	0.227	0.651	0.227	0.134	0.712
**VGG16 (224x224x3)**	23,752,273	0.801	0.499	0.917	0.499	0.417	0.787
**ResNet50 (224x224x3)**	**32,561,114**	0.845	0.803	0.973	0.803	0.803	0.906
**Our model (256x256x3)**	**19,243,317**	**0.863**	**0.802**	**0.972**	**0.802**	**0.798**	**0.908**
